# BRG1 promotes hepatocarcinogenesis by regulating proliferation and invasiveness

**DOI:** 10.1371/journal.pone.0180225

**Published:** 2017-07-12

**Authors:** Benedikt Kaufmann, Baocai Wang, Suyang Zhong, Melanie Laschinger, Pranali Patil, Miao Lu, Volker Assfalg, Zhangjun Cheng, Helmut Friess, Norbert Hüser, Guido von Figura, Daniel Hartmann

**Affiliations:** 1 Department of Surgery, Klinikum rechts der Isar, Technische Universität München, Munich, Germany; 2 II. Medizinische Klinik und Poliklinik, Klinikum rechts der Isar, Technische Universität München, Munich, Germany; 3 Department of General Surgery, the Affiliated Zhongda Hospital, Southeast University, Nanjing, China; University of South Alabama Mitchell Cancer Institute, UNITED STATES

## Abstract

The chromatin remodeler complex SWI/SNF plays an important role in physiological and pathological processes. Brahma related gene 1(BRG1), a catalytic subunit of the SWI/SNF complex, is known to be mutated in hepatocellular carcinoma (HCC). However, its role in HCC remains unclear. Here, we investigate the role of BRG1 on cell growth and invasiveness as well as its effect on the expression of putative target genes. Expression of BRG1 was examined in human liver tissue samples and in HCC cell lines. In addition, BRG1 was silenced in human HCC cell lines to analyse cell growth and invasiveness by growth curves, colony formation assay, invasion assay and the expression of putative target genes. BRG1 was found to be significantly increased in HCC samples compared to non-HCC samples. In addition, a declined proliferation rate of BRG1-silenced human HCC cell lines was associated with a decrease of expression of cyclin family members. In line with a decreased invasiveness of BRG1-siRNA-treated human HCC cell lines, down-regulation of MMP7 was detected. These results support the hypothesis that overexpression of BRG1 increases cell growth and invasiveness in HCC. Furthermore, the data highlight cyclin B, E and MMP7 to be associated with BRG1 during hepatocarcinogenesis.

## Introduction

Liver cancer is the fifth most common cancer in men and seventh most common cancer in women worldwide [1]. Accounting for more than 85%, hepatocellular carcinoma (HCC) is the most common histopathological type of primary liver cancer [2]. A large number of mutations in different genes have been identified in HCC to date [3]. There is growing evidence for the importance of the SWI/SNF chromatin remodeling complex during hepatocarcinogenesis based on the detection of mutations and gene alterations in various subunits of the SWI/SNF chromatin remodeling complex in HCC [4].

Chromatin remodeling complexes modify chromatin structure and regulate transcription of genes to control different cellular processes. Mammalian SWI/SNF chromatin remodeling complexes are the most mutated chromatin regulators in human cancer [5]. Evolutionarily conserved, the mammalian SWI/SNF complexes are separated into two groups: the brahma related gene 1 (BRG1)-associated factor complex (BAF) and the polybromo BRG1-associated complex (PBAF). These two complexes differ in their respective catalytic ATPase subunits. The BAF complex utilises either BRG1 or BRM as the catalytic subunit, whereas the PBAF complex is composed of BRG1 exclusively. In association with these catalytic subunits, various other proteins contribute to the SWI/SNF complexes that are finally composed of 9 to 12 subunits [6,7,8]. The mutational landscape of human cancer reveals different subunits of the SWI/SNF complexes including BRG1 to be frequently mutated and altered [3,4,9,10]. However, the role of mutated BRG1 in tumourigenesis remains largely unknown. Various human cancers reveal an overexpression of BRG1, whereas a similar number of malignant tumours show the suppression of BRG1 expression [11–24]. In addition, BRG1 is known to interact with both proliferation-promoting and -inhibiting genes, including cyclins and pRB [16,17,19,25]. This implies that BRG1 not only acts as a tumour suppressor gene, but also as an oncogene. However, at present it is not clear when BRG1 acts as a tumour suppressor gene and when it acts as an oncogene.

In HCC, BRG1 reveals four different somatic heterozygous, missense mutations, causing overexpression [11]. One of these somatic mutations was found in the catalytic ATPase domain. This domain enables mechanical movement by converting ATP energy. Two somatic mutations were detected in the bromodomain, a domain that is involved in the recognition of acetylated lysines in histone tails [11]. While Endo et al. (2013) [11] observed no correlation in HCC for BRG1 expression and overall survival or any other clinicopathological parameters, Zhu et al. (2016) [12] detected a positive correlation between increased BRG1 expression and the severity of HCC as well as metastasis. Moreover, BRG1 plays an important role in the regulation of liver cancer stem cells [12]. However, the specific role of BRG1 in HCC remains largely unclear at present. In this study, the role of BRG1 on proliferation and invasion in human HCC cancer cell lines was investigated. In addition, target genes regulating the cell cycle and the ability of invasion were analysed. Our findings support the hypothesis that BRG1 promotes proliferation as well as invasion in HCC and highlight the correlation between the expression of BRG1 and members of the cyclin family as well as matrix metalloproteinases.

## Materials and methods

### Cell lines and cell cultures

All cell experiments were performed by using the the human HCC cell line HuH7 and the human hepatoblastoma cell line HepG2 purchased from the Japanese Collection of Research Bioresources Cell Bank/ National Institutes of Biomedical Innovation, Health and Nutrition (JCRB Cell Bank/ NIBIOHN, Osaka, Japan) and the American Tissue Culture Collection (ATCC, Manassas, VA, USA). HepG2 cells were cultured in Dulbecco`s Modified Eagle Medium 4,5% Glucose supplemented with 10% fetal bovine serum and 1% penicillin/streptomycin. HuH7 cells were cultured in Dulbecco`s Modified Eagle Medium 1% Glucose supplemented with 10% fetal bovine serum and 1% penicillin/streptomycin. All cells were cultivated in 5% CO2 and 20% O2 at 37°C.

### Liver tissue

Liver tissue was recruited from the Surgery Department of the Klinikum rechts der Isar, Technical University Munich. Patients undergoing surgery mainly suffered from HCC or liver metastases of an extra hepatic primary tumour. By using liquid nitrogen one part of each tissue sample was quick-frozen and stored at -80°C until required. Another part of each sample was embedded into paraffin to perform immunohistochemistry. A full amount of 36 tissue samples was analysed consisting of 13 HCC tumour samples, 10 non-tumour counterpart samples and 13 non-tumour liver samples from patients mostly suffering from liver metastases of an extra hepatic primary tumour. Non-tumour tissue included non-fibrosis (n = 13) and fibrosis /cirrhosis (n = 10) tissue.

For immunohistochemistry 11 HCC tumour samples were available. All tissue samples used for immunohistochemistry were also analysed for BRG1 expression by qRT-PCR.

The study on human material was approved by the institutional review board of the Medical Faculty of the Technical University of Munich and designed in accordance with the Declaration of Helsinki (Approval number: 1926/07). Written informed consent was obtained from patients.

### Immunohistochemistry

Sections were rehydrated by washing in ethanol and distilled water. For Antigen retrieval the sections were cooked in citrate solution for 15min. Endogenous peroxidase activity was blocked using 3% hydrogen peroxide. After incubating the sections with 0,3% Triton for 10min the sections were incubated with 10% goat serum/0,3% Triton blocking solution for 1h at room temperature. The sections were incubated overnight with the polyclonal rabbit anti-BRG1 antibody (1:100, Santa Cruz, CA, USA) at 4°C. Afterwards, the sections were incubated with the secondary antibody (Dako Envision+ System-HRP Labelled Polymer, Anti-Rabbit) for 1h. For staining the sections diaminobenzidin (DAB) (Liquid DAB+ Substrate Chromogen System, DAKO) was used. To stop the reaction after 1min, the sections were rinsed in distilled water. Finally, the sections were counterstained with haematoxylin and dehydrated again. Staining was scored positive for BRG1 expression if at least 10% of counted cells were positive for BRG1 expression. For a more accurate analysis of the positive BRG1 sections the immunreactive score of Remmele [26] was performed. The score scale considers intensity and percentage of positive stained cells and reaches from 0 to 12 points whereby 12 means the maximum of expression. All sections were analysed by an experienced pathologist.

### Transfection

siRNA: Cells were seeded freshly. After a cell confluence of 30–50%, siRNA transfection was performed using INTERFERin® (Polyplus-transfection) according to the standard protocol described by the manufacturer. Three different siRNAs targeting BRG1 (Ambion®/ Invitrogen™/ Thermo Fisher Scientific) were used. All experiments were carried out with a negative control (Ambion®/ Thermo Fisher Scientific).

1. siRNA-BRG1      Sense          CCU CCG UGG UGA AGG UGU CUU ACA A

                                Antisense    UUG UAA GAC ACC UUC ACC ACG GAG G

2. siRNA-BRG1      Sense            GGU GAU CCA CGU GGA GAG UTT

                                Antisense    ACU CUC CAC GUG GAU CAC CTT

3. siRNA-BRG1      Sense            GGA AUA CCU CAA UAG CAU UTT

                                Antisense    AAU GCU AUU GAG GUA UUC CTG

Plasmid: Cells were seeded freshly. After a cell confluence of 30–50% transfection of pBABE-puro (Addgene plasmid #1959 with excised Brg1) or pBABE-BRG1 (Addgene plasmid #1959, Sif et. al., 2001, [27]) was performed by using FuGENE HD (Promega, USA).

### RNA isolation and quantitative real time PCR analysis

In both human liver tissue and human cell lines, RNA was isolated using the RNeasy Mini Kit (QIAGEN). QuantiTect Reverse Transcription Kit (QIAGEN) was used to synthesize complementary DNA. Quantitative real time PCR was prepared with LightCycler 480 SYBR®Green I Mix (Roche) and performed with the LightCycler 480 (Roche). As an endogenous control for mRNA levels expression was normalized against hypoxanthine-guanine phosphoribosyl transferase (HPRT). The Light Cycler® 480 SW 1.5 software was used to analyse the data. Primers were purchased from Metabion.

### Western blotting

Protein was extracted by using RIPA Buffer according to the manufacturer’s standard protocol (Cell Signaling Technology). Determination of the concentration of proteins was performed by using the Pierce™ BCA Protein Assay Kit (Thermo Scientific) following its protocol. After gel electrophoresis and blotting on a Whatmann Protran BA85 membrane (GE Healthcare), membranes were incubated with primary antibody rabbit anti-BRG1 (1:2500, Santa Cruz) overnight. Incubation with a secondary antibody anti-Rabbit (1:2000, Promega) for 1h was followed by developing the membranes using ECL™ Western Blotting Detection (GE Healthcare) as detection solution.

### Growth curves

Proliferation was determined by performing growth curves using the Neubauer-improved counting chamber (Marienfeld) to count siRNA transfected and untransfected cells. After siRNA transfection was performed, an equal number of cells were seeded for each group of cells treated differently. As soon as the first group of cells of HepG2 or HuH7 cell line reached a cell confluence of 80–90%, all groups of the cell line were counted and seeded again in an equal number of cells. All groups of cells of each cell line were seeded twice, one to continue the growth curve, one to analyse BRG1 expression at different time points.

Growth curves for the cells transfected with a plasmid were performed by counting the cells with the Countess II Automated Cell Counter (ThermoFisher, USA) 3 days and 5 days after transfection.

### Colony formation assay

Transfection was performed as mentioned previously. 48h after transfection an equal number of cells was seeded and cultured for 2 weeks. The cultured medium was refreshed every 4 days. After washing the cells with PBS three times the resulting cells were then fixed in methanol and stained with crystal violet. Individual colonies with more than 50 cells were counted.

### 3-(4,5-dimethylthiazol-2-yl)-2,5-diphenyltetrazolium bromide (MTT) assay

3-(4,5-dimethylthiazol-2-yl)-2,5-diphenyltetrazolium bromide (MTT) assay was used to determine cell proliferation. After transfection of the plasmids to induce BRG1 overexpression cells were seeded freshly. MTT reagent (Roth, Germany) was added to the samples. Following 4h of incubation at 37°C, the medium was carefully removed and the intracellular formazan products were lysed by addition of Dimethylsulfoxid (Roth, Germany). The absorbance was measured at 570nm using a microplate reader (Promega, WI, USA).

### Flow cytometry

24h hours after transfection, cells were synchronized by serum starving (0% serum) for another 24h. Next, incubation in Dulbecco`s Modified Eagle Medium 1% Glucose for 1h was followed. Subsequently, cells were harvested by trypsinization, centrifuged and fixed in 70% ethanol for more than 30 minutes at 4°C. Cells were stained and treated with propidium iodide (Becton Dickinson, San Jose, CA, US) and RNase A (Sigma-Aldrich, MO, US). BD FACSCanto flow cytometer (BD Biosciences, San Jose, CA, US) was used for analyses. Cell distribution in the different phases of the cell cycle was analysed using FlowJo analysis software (Tree Star, Ashland, OR, US). Gating was applied to exclude cell debris, cell doublets, and cell clumps. For positive control the cells were incubated with nocodazole in Dulbecco`s Modified Eagle Medium 1% Glucose for 6h before collection.

### Invasion assay

Cell invasion assay was performed using Matrigel Invasion Chambers (Corning) as described by the manufacturer. Cells were seeded in equal numbers in upper chambers with 8.0μm pore, HepG2 cells in serum free medium, HuH7 cells in medium containing 1% bovine serum. Medium supplied with 20% bovine serum acted as chemoattractant. After 44h of incubation, non-invading cells were removed from the upper surface. Invaded cells were fixed and stained with triphenylmethane dye. A microscope with a 20x magnification was used to count five randomly chosen power fields.

### Statistical analysis

Data are presented as means ± standard deviation. All experiments were performed in triplicates at least. Student’s t-test was used to determine P values. P < 0.05 was considered as statistically significant. Microsoft Excel and GraphPad Prism software were used to perform statistical analyses.

### Repeatability of experiments

The following experiments growth curves, colony formation assay, invasion assay, MTT assay and target gene expression analysis were performed at least two times independently. Experiments using transfection of siRNA were performed with at least two different siRNAs targeting BRG1 and a negative control all time. Shown are representative data for each experiment.

## Results

### BRG1 expression is upregulated in HCC

To examine BRG1 expression in HCC and non-tumour liver tissue, different tissue samples from patients undergoing liver surgery were analysed. In total, 36 specimens consisting of HCC tissue (n = 13), non-tumour tissue counterparts (n = 10) and non-tumour liver tissue of patients not suffering from HCC (n = 13) were included in this study and analysed by qRT-PCR. Furthermore, an accurate investigation of varying BRG1 expression within different HCC samples was performed by immunohistochemistry.

**Fig 1:**
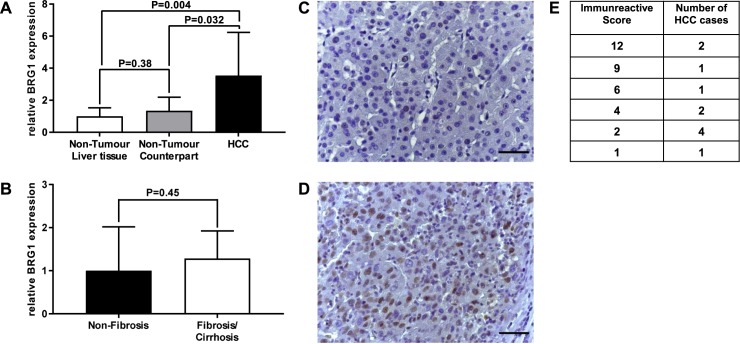
Analysis of BRG1 expression in HCC by qRT-PCR and immunohistochemical staining. (**A,B**) BRG1 is overexpressed significantly in HCC tissue (n = 13) compared to non-tumour counterpart (n = 10) respectively non-tumour liver tissue of patients not suffering from HCC (n = 13). A more detailed analysis of non-tumour liver tissue revealed no difference of BRG1 expression between tissue of non-fibrosis (n = 13) and fibrosis/cirrhosis (n = 10). Shown are the relative expression levels, non-tumour liver tissue respectively non-fibrosis tissue were standardised as 1. (**C**) Normal hepatocytes are showing no expression of BRG1. (**D**) Positive BRG1 staining in HCC tissue. (**G**) Analysis of BRG1 expression in immunohistochemistry by immunoreactive score. A varying degree of BRG1 expression in HCC was found ranging from a minor score of 1 to a maximum score of 12. The determined scores were evenly distributed. Differences were due to a high variety of intensity as well as the percentage of positive stained cells. Scale bar represents 50μm.

At first, mRNA levels of BRG1 from all patient tissues included in the study were analysed by qRT-PCR. In HCC tissue, BRG1 was found to be significantly overexpressed compared to non-tumour liver tissue of patients not suffering from HCC (P = 0.004) (Fig 1A). HCC tissue also exhibited a significant increase in BRG1 expression compared to non-tumour tissue counterparts (P = 0.032) (Fig 1A). There was no difference between non-tumour tissue counterparts and non-tumour liver tissue of patients not suffering from HCC (P = 0.38) (Fig 1A). In addition, a more detailed analysis of all non-tumour samples revealed no difference between the two groups of non-fibrosis and fibrosis/cirrhosis (Fig 1B).

**Fig 2:**
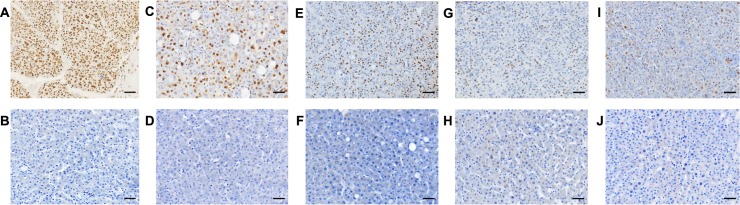
Immunohistochemical staining of BRG1 in HCC. **(A-J)** BRG1 is expressed in HCC tissue **(A,C,E,G,I)** but not in non-tumour tissue counterpart **(B,D,F,H,J)**. **(A,C,E,G,I)** HCC tissue is showing different expression levels of BRG1 due to a high variety of intensity as well as the percentage of positive stained cells. Scale bar represents 50μm.

Next, the protein levels of BRG1 expression were determined by immunohistochemistry (Figs 1C, 1D and 2A–2J). The findings from this study confirmed a previous report by Endo et al. (2013) [11], which showed that typically BRG1 is not expressed in the nuclei of normal hepatocytes but in the nuclei of bile duct epithelial cells and in malignant transformed HCC cells (Figs 1C, 1D and 2A–2J). HCC tissue was considered positive for BRG1 expression if more than 10% of cells showed a positive staining. Based on that evaluation score, 91% (10 out of 11) of all HCC samples were positive for protein expression of BRG1. A more detailed analysis of the expression of BRG1 was performed by applying an immunoreactive score. A varying degree of BRG1 expression was found in HCC, ranging from a minor score of 1 to a maximum score of 12 (Fig 1E). These differences were due to a high variety of intensity as well as the percentage of positive stained cells (Fig 2A, 2C, 2E, 2G and 2I).

### BRG1 knockdown impairs proliferation and modulates cyclin family

**Fig 3:**
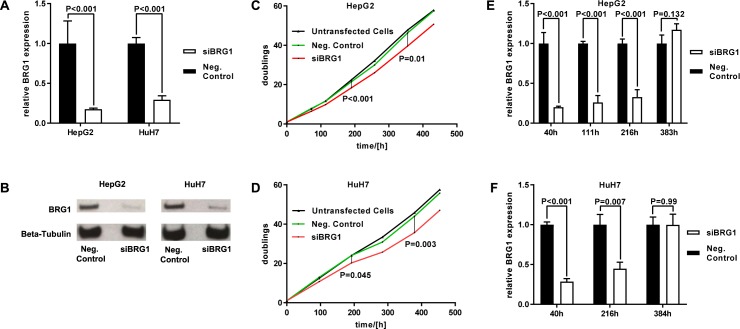
BRG1 knockdown impairs proliferation. (**A,B**) Human HCC cell lines HepG2 and HuH7 showed expression of BRG1 on mRNA and protein level. (**A**) After transfection, a highly significant down-regulation of mRNA levels of BRG1 was achieved for both HepG2 (P<0.001) and HuH7 (P<0.001) cell lines, n = 4. (**B**) Analysed by Western Blot, protein expression was also decreased in HepG2 and HuH7 cell lines after transfection, n = 3. Proliferation was analysed by growth curves for both human HCC cell lines HepG2 and HuH7. (**C,D**) Growth curves, n = 3 and (**E,F**) BRG1 expression at different time points after transfection, n = 3. Growth curves of HepG2 (**C**) and HuH7 (**D**) cells revealed a significant decrease of proliferation as long as BRG1 is suppressed significantly (**E,F**). 10 days after transfection proliferation rates began to equalize (**C,D**) and siRNA targeting BRG1 started to lose its impact (**E,F**). (**E,F**) Negative control was standardised as 1.

To determine the effects of BRG1 on proliferation and invasion, human HCC cell lines HuH7 and HepG2 (hepatoblastoma), expressing BRG1 (Fig 3A and 3B), were analysed. First, it was found that transfection with siRNA targeting BRG1 leads to a sufficient knockdown of BRG1 by analysing mRNA and protein levels of BRG1. For both cell lines the highly significant (p<0.001) suppression of BRG1 expression was determined by qRT-PCR (Fig 3A). Examination of protein levels by Western blot also confirmed an efficient knockdown of expression of BRG1 for both cell lines (Fig 3B).

**Fig 4:**
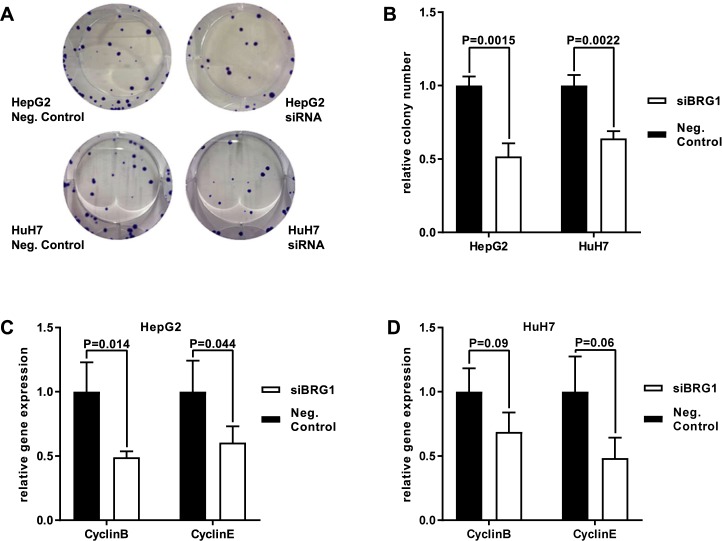
BRG1 knockdown impairs colony formation and modulates cyclin family. (**A,B**) Colony formation assay was performed to analyse proliferation of both human HCC cell lines HepG2 and HuH7. Colony formation was reduced significantly in both cell lines, HepG2 and HuH7, n = 3. (**C**) Analysis of mRNA expression by qRT-PCR showed a significant down-regulation of cyclinB and cyclinE expression in BRG1-suppressed HepG2 cells at the time point of 40h after transfection, n = 4. (**D**) HuH7 cells showed a trend towards lower expression though this was not significant, n = 4. Negative control was standardised as 1.

The next step was to investigate cell proliferation after the suppression of BRG1 and to analyse genes linking BRG1 to a proliferative effect. To address the effect of BRG1 knockdown on proliferation, growth curves were performed. In addition, the expression of BRG1 was analysed at different time points to ensure a sufficient knockdown of expression of BRG1 and to detect the time point at which knockdown declined. For both cell lines, a significant decrease in cell growth for cells transfected with siRNA targeting BRG1 was found for the first 10 days after transfection. After 10 days, proliferation rates of all three tested cell groups of each cell line started to equalize (Fig 4C and 4D). The analysis of expression of BRG1 showed a significant knockdown for the first 9 days after transfection. Sixteen days after transfection in both cell lines, the expression level of BRG1 was equalized in all three cell groups again (Fig 3E and 3F). Taken together, these data point out that the suppression of BRG1 reduces cell proliferation in human HCC cell lines.

Performing a colony formation assay was a different way to determine the role of BRG1 on proliferation. Therefore, each cell line was transfected with a negative control and siRNA targeting BRG1. Analysing cell cultures over 14 days revealed a significant reduction in the number of colonies as well as a smaller size of colonies in BRG1-silenced cells. In both cell lines, HepG2 and HuH7, the ability of colony formation decreased by about 40–50% (Fig 4A and 4B) These findings support the data obtained from growth curves, indicating that BRG1 promotes proliferation.

Induced overexpression of BRG1 in the HuH7 cell line by transfection of a plasmid carrying the BRG1 gene was used to specify the role of BRG1 on proliferation in HCC. Performing growth curves and MTT assay after transfection of the plasmid showed a significant increase of proliferation in induced BRG1 overexpressed cells (S1 Fig).

Next, different cell cycle genes were examined by qRT-PCR after BRG1 knockdown. Again, both cell lines were transfected with a negative control and siRNA targeting BRG1. Members of the cyclin family were analysed at a time point of 40h after transfection. For cyclin B and cyclin E, a significant decrease in mRNA expression levels was detected in HepG2 cells transfected with siRNA targeting BRG1 (Fig 4C). HuH7 showed a trend towards lower expression, although this was not significant (Fig 4D).

To address the question whether BRG1 might reduce proliferation by inducing cell cycle arrest we performed flow cytometry analysis. Therefore, HuH7 cell line was analysed 48h after transfection by flow cytometry. In BRG1-silenced cells the cell population in G2 phase showed a significant increase whereas the cell population in G1 phase was significantly decreased (S2 Fig).

Taken together, these data point to BRG1 as an important mediator for proliferation and modulation of the cyclin family.

### BRG1 knockdown impairs HCC invasion and modulates MMP7

**Fig 5:**
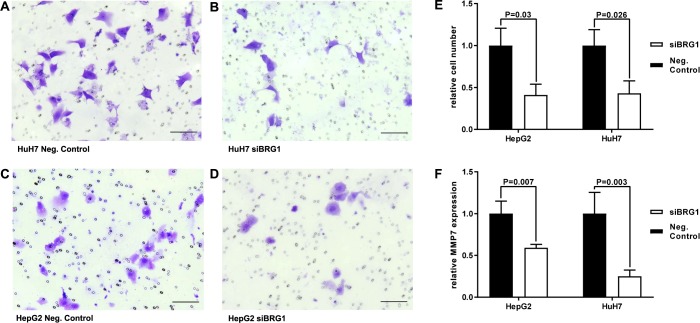
BRG1 knockdown impairs invasiveness and modulates MMP7. Invasion assay was performed to analyse the role of BRG1 for invasive ability of human HCC cell lines. (**A-E**) In both cell lines down-regulated BRG1 expression impaired invasive ability. (**F**) Analysis of mRNA levels after down-regulation of BRG1 showed a significant decrease of MMP7 expression for both cell lines 40h after transfection. Negative control was standardised as 1. Scale bar represents 100μm.

The question of whether BRG1 has an impact on the invasion of HCC cells was addressed next. For this purpose, invasion assays were performed for both HepG2 and HuH7 cell lines. In accordance with previous investigations of this study, BRG1 expression was knocked down in both cell lines using siRNA targeting BRG1. Then, 44h after seeding the cells on chambers membrane, an analysis of invaded cells was performed. The data showed that the suppression of BRG1 compromises the invasive ability of both HepG2 and HuH7 cells significantly by more than 50% (Fig 5A–5E). To elucidate the invasive reduction of BRG1-down-regulated cells, mRNA expression of MMP7 as a member of the matrix metalloproteinases (MMP) family was analysed by qRT-PCR. MMPs are key proteases for the remodelling of the extracellular matrix and are closely linked to the invasiveness of cells. Analysis of the data of this study demonstrated that the expression of MMP7 decreased significantly in BRG1-down-regulated cells of both HepG2 and HuH7 cell lines (Fig 5F). In summary, these data reveal that BRG1 suppression impairs invasion, whilst causing a decline of MMP7 expression.

## Discussion

A number of previous studies have shown alterations of BRG1 expression in various human cancers. However, BRG1 appears to play a contrasting role for tumourigenesis [11–24]. For HCC, different somatic missense mutations were reported, as well as an increased expression [11]. However, to date the role of BRG1 in HCC remains unclear. In this study, an overexpression of BRG1 in HCC has been shown to promote proliferation and invasion. In addition, a modulating effect for members of the cyclin family and MMP7 by BRG1 was detected, which may be an important link between BRG1 and proliferation as well as invasion.

BRG1 overexpression is known in a various number of malignant tumours [11–17,19,20,24]. Endo et al. (2013) [11] demonstrated that BRG1 is overexpressed in HCC compared to non-tumour tissue counterparts. No difference was found in BRG1 expression between normal liver, chronic hepatitis and cirrhosis [11]. The first issue of this analysis was to expand those data by determining BRG1 expression in liver tissue from patients not suffering from HCC. It was demonstrated that BRG1 is overexpressed in HCC compared to non-tumour liver tissue of patients not suffering from HCC. In addition, a previous report [11] that showed overexpression of BRG1 in HCC compared to non-tumour counterparts was confirmed. No difference was found in BRG1 expression within the non-tumour liver tissue groups of non-fibrosis and fibrosis/cirrhosis. As both our data and a previous report [11] showed no difference in BRG1 expression within the groups of normal liver, non-fibrosis, chronic hepatitis and fibrosis/cirrhosis, this confirms that alterations of BRG1 may occur at a later time point of the cirrhosis-carcinoma sequence. Interestingly, more than 90% of HCCs investigated by immunohistochemistry revealed a positive staining for BRG1. In contrast, BRG1 was previously reported to be expressed in only 35% of all cases of HCC [11]. In addition, in our study a full range of protein expression of BRG1 in HCC was detected. Similar findings of a distinct level of BRG1 expression in HCC were reported by Zhu et al. (2016) [12]. For breast cancer, a varying expression of BRG1 analysed by immunohistochemistry is also known [17]. Furthermore, varying alterations or expression levels of BRG1 are reported for different subtypes of ovarian cancer and lung cancer [18,21].

For the first time this study showed that BRG1 promotes proliferation in HCC. After the knockdown of BRG1 in both human HCC cell lines, the proliferation rate decreased in growth curves as well as colony formation assays. In previous studies, similar effects of BRG1 on proliferation rate were detected for other human cancers, including colorectal carcinoma, breast cancer, glioma and melanoma [14,16,17,19,24]. A number of studies revealed the interaction of BRG1 and cyclins, which can be either via a direct binding to the promotor or an indirect manner, as the main cause of the reduction of proliferation after silencing BRG1. In these studies, down-regulated BRG1 expression involved a decrease in the expression of cyclin family members to decrease proliferation and induce cell growth arrest [16,17,19,24,28,29]. Our data support this hypothesis, since it was demonstrated that BRG1 modulates cyclin B and cyclin E in HCC, which engage in cell cycle and proliferation.

In addition, BRG1 not only promotes proliferation but also invasive ability. Data from previous studies point out that BRG1 correlates with cell invasion in different types of cancer. It is reported that the overexpression of BRG1 in cells of prostate cancer, melanoma, breast cancer and glioma is associated with cell invasion [16,17,20,30]. In cancer cell lines of melanoma, breast cancer and glioma, the knockdown of BRG1 resulted in a decrease of invasive ability [16,17,30]. In our study, it was demonstrated that the knockdown of BRG1 impairs invasive ability in HCC cell lines. To gain invasive ability, up-regulation of matrix metalloproteinases (MMP) (which interact with the surrounding extracellular matrix) plays a key role in malignant transformed cancer cells. BRG1 is known to interact with MMPs to promote invasion. Previous studies showed that BRG1 directly interacts with the promotor of MMP7 and MMP2 [30,31,32]. Therefore, the modulation of MMP7 by BRG1 in HCC cell lines was investigated. Our observations clearly demonstrate a decrease in MMP7 expression after the down-regulation of BRG1. Recent studies investigating MMPs in the context of cellular invasion of malignant transformed cells revealed a positive modulation of several MMPs by BRG1 including cells of melanoma, breast cancer and glioma [16,17,30,31]. Our data and the findings of previous studies underline the role of BRG1 to promote cellular invasion and modulate MMPs.

In contrast to the hypothesis that BRG1 promotes proliferation in human cancer, re-induction of BRG1 also enables growth arrest within human cancer cell lines lacking BRG1 [22,33]. Therefore, interacting with pRB and cyclin-dependent kinase inhibitors, BRG1 acts as a tumour suppressor gene [33–35]. The factors defining whether BRG1 deals as a tumour suppressor or an oncogene warrant further investigation. Detailed studies of pancreatic cancer formation indicate an important role for the cellular origin of cancer progenitors and distinct stages of cancer formation whether BRG1 acts as a tumour suppressor or an oncogene [32,36]. This implies that BRG1 may function as a context-dependent mediator in tumourigenesis that probably involves the cellular origin as well as the mutational landscape.

In conclusion, this study provides the first evidence that BRG1 influences proliferation as well as invasion in HCC. We revealed an overexpression of BRG1 in HCC and demonstrated that down-regulation of BRG1 impairs proliferation along with the decreased expression of cyclin B and cyclin E. In addition, in line with a decreased invasive ability, a down-regulation of MMP7 was found upon knockdown of BRG1 expression. These findings contribute to a better understanding of BRG1 in HCC and underline the oncogenic function of BRG1 in this context.

## Supporting information

S1 Fig BRG1 overexpression promotes proliferation. (**A**) RT-PCR of BRG1 in HuH7 cell line after transfection with a control vector or a vector containing BRG1. BRG1 was significantly overexpresses in human HCC cell line HuH7 after transfection of the plasmid pBABE-BRG1 compared to the negative control pBABE-puro, n = 5, pBABE-puro was standardised as 1. (**B**) Growth curve of HuH7 cell line. BRG1 overexpressed cells showed a significant higher cell proliferation compared to the negative control pBABE-puro, n = 4. (**C**) MTT assay showed a significant higher proliferation rate in BRG1 overexpressed HuH7 cells, n = 4.
(EPS)

S2 Fig FACS analysis of BRG1 knockdowned cells. (**A**) FACS analysis of HuH7 cell line. After knockdown of BRG1 by transfection of siRNA targeting BRG1 cell population in G2 phase increased while cell population in G1 phase decreased compared to negative control, n = 3.
(EPS)
